# Cytochrome-P450 enzymes and autoimmunity: expansion of the relationship and introduction of free radicals as the link

**DOI:** 10.1186/1740-2557-6-4

**Published:** 2009-06-25

**Authors:** MR Namazi

**Affiliations:** 1 Medicinal and Natural Chemistry Products Research Center and Dermatology Department, Shiraz University of Medical Sciences, Shiraz, Iran

## Abstract

The Cytochrome-P-450 enzymes (CYP) are among the most important xenobiotic-metabolizing enzymes, which produce reactive oxygen species (ROS) as the result of metabolizing xenobiotics.

ROS are believed to play important roles in the pathophysiology of autoimmune diseases. ROS can alter the structure of cellular antigens to produce a "neo-antigen" which could mount an autoimmune response against the original antigen through molecular mimicry. ROS are involved in apoptosis, activation of antigen presenting cells and initiation or amplification of diverse immunologic reactions.

Taking all these facts together, it could be speculated that CYP may be involved in the initiation and/or amplification of autoimmune phenomena.

## 

"Who is in me, heart-weary, now I know not:

While I am mute, a voice within me roars..."

Hafez Shirazi (a great Persian Poet)

## Background

Involvement of cytochrome P450 (CYP) enzymes in the pathogenesis of autoimmune hepatitis type 2, occurring via molecular mimicry of human cytochrome P450 by hepatitis C virus at the level of cytotoxic T cell recognition, is well appreciated [1]. In addition, two different cytochrome P450 enzymes are believed to be the adrenal antigens in autoimmune polyendocrine syndrome type I and Addison's disease [2]. However, except these two diseases where CYP serves as the autoantigen and hence functions as the core of the autoimmunity, the potential contribution of CYP in autoimmune diseases has not been investigated. It is attempted in this paper to draw the attention of the readers to the ability of CYP to induce/amplify autoimmunity through production of free radicals.

### Presentation of the hypothesis

#### a) Brief description of CYP enzymes and their involvement in the production of reactive oxygen species (ROS)

Upon entering the body, a foreign compound is subjected to metabolism by a large group of enzymes, collectively referred as xenobiotic-metabolizing enzymes. Although originally thought to be responsible for drug metabolism almost exclusively in the liver, it has now been realized that all xenobiotic-metabolizing enzymes participate in many crucial endogenous functions, probably in every eukaryotic cell and many prokaryotes. The CYP enzymes are among the most important xenobiotic-metabolizing enzymes and are the products of the CYP superfamily of genes [3]. They are embedded in the phospholipids bilayer of the endoplasmic reticulum [4].

CYPs are named with the root CYP followed by a number designating the family, a letter denoting the subfamily, and another number designating the CYP form. Thus, CYP3A4 is family 3, subfamily A, and gene number 4. All CYPs contain a molecule of heme that is noncovalently bound to the polypeptide chain. Metabolism of a substrate by CYP consumes one molecule of molecular oxygen and produces an oxidized substrate plus a molecule of water as a by-product. However, for most CYPs, depending on the nature of the substrate, the reaction is "uncoupled", consuming more O_2 _than the metabolized substrate and producing activated oxygen or O_2_^- ^[4]. CYPs metabolize most clinically used drugs and are required for metabolic activation of chemical carcinogens and toxins [5]. Additionally, CYPs are involved in the synthesis of endogenous compounds such as steroids and the metabolism of bile acids, which are degradation by-products of cholesterol. Some CYPs, such as those that catalyze steroid and bile acid synthesis, have very specific substrate preferences [4].

The liver contains the greatest abundance of xenobiotic-metabolzing CYPs. More than 50 individual CYP have been identified in humans, of which 12 are known to be important for metabolism of xenobiotics. The expression of different CYPs can differ markedly through interindividual changes resulting from heritable polymorphic differences in gene structure. Several human CYP genes exhibit polymorphisms, including CYP2A6, CYP2C9, CYP2C19, and CYP2D6 [4].

**Figure 1:**
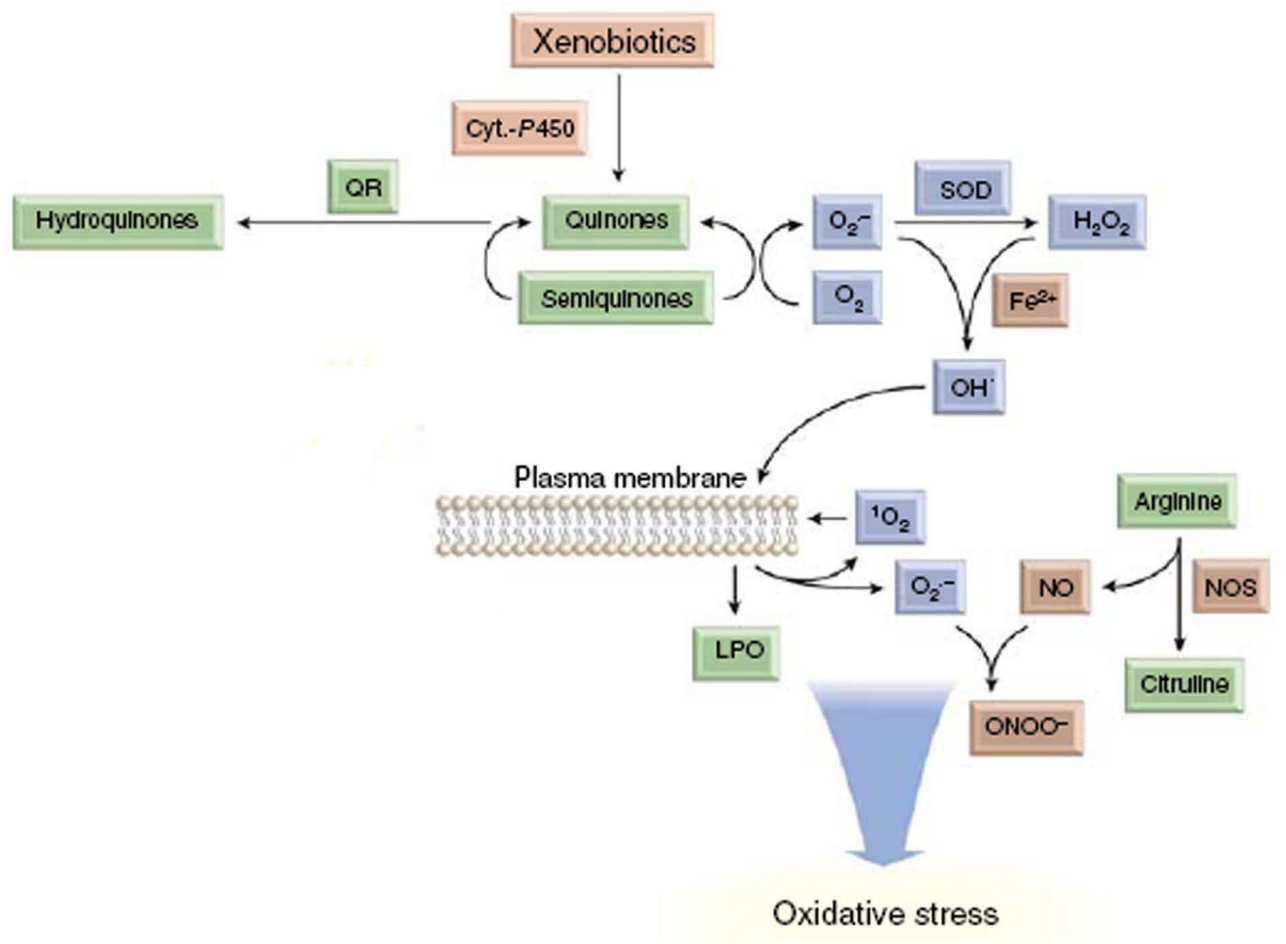
**Generation of ROS by CYP**. Cells generate ROS such as superoxide anion (O_2_^.-^) and H_2_O_2 _as a result of metabolism of xenobiotics by CYP. Both O_2_^.- ^and H_2_O_2 _may be converted to the highly reactive hydroxyl radical (OH^.-^) by iron (Fe^2+^)-catalyzed Haber-Weiss and Fenton reactions. Many xenobiotics are converted to toxic quinones by CYP. These quinones are redox-sensitive agents and are reversibly reduced to semihydroquinones/hydroquinones, which generate O_2_^.-^.

Many xenobiotics are converted to toxic quinones by CYP enzymes (Figure 1). These quinones are redox sensitive agents and are reversibly reduced to semihydoquinones/hydroquinone, which generate superoxide anion. Both superoxide anion and hydrogen peroxide may be converted to hydroxyl radical by iron (Fe^2+^)-catalyzed Haber-Weiss and Fenton reactions [6]. Theses reactive molecules are more often derived from foreign chemicals (for example, insecticides) than from endogenous substrates (for example, lipid peroxides) [7].

#### b)The important role of ROS in the pathogenesis of autoimmunity

There are several ways by which ROS could contribute to the development of autoimmunity. These mechanisms, also discussed fully in reference [9], are as follows:

The structures of cellular macromolecules and small molecules may markedly change by acute or chronic oxidative stress, acting as antigens ("neo-antigens"). Neo-antigens with sufficient homology or identity to host antigenic proteins induce auto-reactivity. This phenomenon is referred to as "molecular mimicry" [8,9].

Aldehydic products, mainly the 4-hydroxy-2-alkenals, form adducts with proteins and make them highly immunogenic [10]. Hydroxyl radicals are also very highly reactive and could attack a wide range of targets. The presence of rheumatoid factors in some autoimmune diseases, such as vitiligo [9,11] and rheumatoid arthritis, can be explained by this mechanism. Over time, chronic oxidative stress could generate several adducted and/or non-adducted molecules that would essentially act as a "neo-antigens". This is consistent with the slow maturation of auto-antibodies in the evolution of autoimmune diseases. During chronic oxidative stress, neo-antigens potentially cause tissue damage and release a plethora of sequestered auto-antigens. This process is referred to as the "bystander effect". Such an outburst of auto-antigens from the target tissue would potentially amplify the effect of the neo-antigens, leading to the breakdown of self-tolerance [8].

Reactive oxygen species are recognized as important signalling molecules within the cells of the immune system. This is, at least in part, due to the reversible activation of kinases, phosphatases and transcription factors by modification of critical thiol residues [9,12]. In fact, free radicals are involved in specific early events in T cell activation and antioxidants reduce T cell proliferation, IL-2R expression and IL-2 production [13].

It was recently reported that ROS upregulate dendritic cell surface markers, including MHC Class II molecules, suggesting that antigen-specific, bidirectional dendritic cell-T-cell communication can be blocked by interfering with redox regulation pathways. ROS play a crucial role in activation of sentinel dendritic cells, linking tissue damage to initiation of an immune response [9,14]. Addition of DNFB, a strong skin sensitizer, to a dendritic cell line generated from fetal mouse skin enhanced protein oxidation and induced p38 MAPK and extracellular signal-regulated kinase (ERK)1/2 phosphorylation, which could be blocked by GSH [15].

Reactive oxygen species activate NF-κB through activation of kinases [16]. On activation, NF-κB regulates the expression of almost 400 different genes, which include enzymes such as iNOS, cytokines (such as TNF-α, IL-1 and chemokines), and adhesion molecules [9,17].

Oxidative processes enhance the reaction of the adaptive response. Oxidation of carbohydrates enhances the antibody response to coadministered coantigens. Moreover, the administration of the Schiff base-forming agent tucaresol during immunization with protein antigen increased T-cell-dependent immune response. Direct modification of protein antigen has been demonstrated to be required for the enhancement of the immune response [9,18].

Oxidative stress, which can induce apoptosis by releasing caspase activating cytochrome C from mitochondria [19], may induce or contribute to apoptosis. Apoptosis is believed to be involved in autoimmunity. During apoptosis, modification of cellular antigens through proteolysis, changes in the phosphorylation state and citrullination may give rise to potentially immunostimulatory forms of intracellular or membrane-associated autoantigens. Generally, the efficient clearance of apoptotic cells results in the exposure of intracellular self-antigens to the immune system under non-inflammatory conditions, leading to tolerizing of these antigens. It has been proposed that under these conditions circulating dendritic cell precursors take up apoptotic cells and travel to lymphoid organs, where they present autoantigens from apoptotic cells to T cells in the absence of costimulatory molecules. However, under a proinflammatory environment, these modified autoantigens, which may also expose cryptic epitopes, may be processed by mature Langerhans' cells and presented to either naïve T cells that have not been tolerized against the cryptic epitopes or to autoreactive CD4+ and CD8+ that escaped deletion due to defects in T cell apoptosis. Subsequently the autoreactive CD4+ T cells may stimulate autoreactive B cells to produce autoantibodies, whereas CD8+ T cells may attack cellular antigens directly. It deserves noting that efficient clearance of apoptotic cells is crucial for the avoidance of autoimmune responses to intracellular antigens [9,20].

In sum, oxidative stress plays an important role in the etiopathogenesis of the autoimmune diseases by initiating or amplifying the autoimmune response.

## Conclusion

Given that the metabolism of xenobiotics by CYP leads to the production of ROS, and that ROS contribute crucially to the initiation and/or amplification of the autoimmune response, CYP may play a role in the pathobiology of some autoimmune diseases.

The author wishes to put more emphasis on the potential link between CYP polymorphisms and vitiligo, as vitiligo is an autoimmune disorder in which not only ROS but also quinones, which could be produced by CYP, are supposed to be crucially involved. It is supposed that reactive quinones can be covalently bound to the catalytic centre of tyrosinase to give a neo-antigen. Micro-molar (noncytotoxic) quantities of *o*-quinones may be sufficient in this haptenation to mount an immune response [21].

As mentioned, some cytochrome P450 (CYP) heme-thiolate enzymes participate in the detoxication of xenobiotics. Paradoxically, they can produce reactive intermediates of thousands of chemicals that can damage DNA, as well as lipids and proteins. CYP expression can also affect the production of molecules derived from arachidonic acid, and alter various downstream signal-transduction pathways. Such changes can be precursors to malignancy. It is thus believed that CYP could play role in environmental carcinogenesis [22]. Several studies have indicated a link between rheumatic diseases, autoimmune phenomena, and cancers. An increased risk of hematological malignancies, compared with the general population, was found among patients with rheumatoid arthritis and systemic lupus erythematosus. Similarly, the prevalence of solid tumours among patients with systemic sclerosis is between 3 and 7%. Cancer is also common among dermatomyositis patients [23]. It is suggested that the link between autoimmune phenomena and rheumatic diseases may be a result of (a) generation of autoantibodies against various autoantigens, (b) paraneoplastic syndromes, (c) rheumatism after chemotherapy, a clinical entity characterized by the development of musculoskeletal symptoms after combination chemotherapy for malignancy. I would like to suggest that the involvement of CYP in both carcinogenesis and autoimmunity may prove to be a hitherto unexplained reason for association between cancer and autoimmunity.

## Testing the hypothesis

The hypothesis can be tested by comparing the frequency of poor metabolizers, i.e. those with genetically determined low or no CYP activity, to extensive metabolizers, i.e. those having fully functional CYP activity, in diverse autoimmune diseases.

## Implications of the hypothesis

If proven, a practical implication of this hypothesis is the prevention of occurrence of autoimmune diseases in predisposed individuals or the prevention of relapse of the autoimmune disease in affected individuals by CYP inhibitors.

## Abbreviations

CYP: Cytochrome-P-450 enzymes; MAPK: Mitogen-activated protein kinase; NF-κB: Nuclear factor kappa-light-chain-enhancer of activated B cells; ROS: Reactive oxygen species

## Competing interests

The author declares that they have no competing interests.
